# Abdominal wall endometriosis: A challenging iatrogenic disease^☆^

**DOI:** 10.1016/j.ijscr.2021.106507

**Published:** 2021-10-14

**Authors:** Mohamed Aymen Ferjaoui, Ramzi Arfaoui, Slim Khedhri, Kais Abdessamia, Mohamed amine Hannechi, Kmar Hajjami, Raja Azri, Manel Benghalia, Monia Malek, Mohamed Radhouane Rachdi, Khaled Neji

**Affiliations:** a Department B of Gynecologic Surgery, Tunis Maternity Center, Tunis Medical School, el Manar University, Tunisia; b Maternity Department of Tunis Military Hospital, Tunis Medical School, el Manar University, Tunisia

**Keywords:** Endometriosis, Abdominal wall, Caesarean section, Gynaecologic surgery

## Abstract

**Introduction and importance:**

Abdominal wall endometriosis is a rare clinical condition associated with abdominal pain and psychologic disorders. It's pathophysiology remains unclear. Clinical history and imaging findings are necessary for the diagnosis. Its management is challenging, and requires close collaboration between gynaecologists and visceral surgeons specially in complex procedures. The aims of our study are to present risk factors, clinical presentation, imaging findings and management features. It was a retrospective descriptive study including fifteen patients presenting abdominal wall endometriosis. Data about age, medical history, imaging findings, surgical procedures and outcome are reported.

**Cases presentation:**

Fifteen women were included in our study. The most common symptom was cyclic abdominal pain. Twelve of them had history of caesarean section, and three had history of myomectomy. All patients underwent ultrasound and MRI. We performed surgical excision to all cases. One patient needed large excision with abdominoplasty procedure.

**Clinical discussion:**

Abdominal wall endometriosis is a rare clinical condition with unclear pathophysiology. It occurs frequently after gynaecologic or obstetric surgery. Most reported complaint was catamenial abdominal pain with abdominal wall mass. Ultrasonography, computed tomography and MRI are useful for diagnosis, specially to eliminate differential diagnoses. Abdominal wall endometriosis management is based on surgery. Excision goals are to remove the mass and to confirm histological diagnosis of parietal endometriosis.

**Conclusion:**

Parietal endometriosis is a rare and challenging condition with unclear pathophysiology. It requires specific management. This pathology will be encountered more frequently considering the increasing rate of caesarean section.

## Highlights

• Extra pelvic endometriosis locations are rare, especially in the abdominal wall.
• It is a challenging condition with an important social and medical impacts caused by symptoms.
• We present a multicentric Tunisian experience of abdominal wall endometriosis management.

## 1 Introduction

Endometrial tissue migration is commonly located in ovaries, pelvic peritoneum, gastrointestinal and urinary tracts. Abdominal wall endometriosis is a rare condition. It is characterized by the development of ectopic endometrial cells and stroma in abdominal wall layer. It occurs commonly after surgical procedures such as caesarean section or myomectomy [1]. The incidence of abdominal wall endometriosis is increasing because of the high rate of caesarean section [1].

This challenging and rare condition is associated with chronic pelvic pain and psychologic disorders.

In our study, we reviewed all cases of abdominal wall endometriosis in the last three years and managed in two gynaecologic surgery centers: Department B of gynaecologic surgery of Tunis maternity center and Maternity department of Tunis military hospital. Our study aims to assess clinical, diagnostic and therapeutic features of abdominal wall endometriosis. The work has been reported in line with the SCARE criteria [2].

## 2 Materials and methods

It's a retrospective and observational case series study of patients with abdominal wall endometriosis managed in department B of gynaecologic surgery and obstetrics of Tunis maternity center and maternity department of Tunis military hospital. This study was conducted from January 2018 to December 2020. Fifteen patients with abdominal wall endometriosis were reported. Medical records regarding age, medical history, clinical presentation, imaging findings, surgery and postoperative outcome were collected and analysed. All surgical procedures were performed by senior gynaecologist seniors.

## 3 Results

Fifteen patients were enrolled in our study. The mean age was 32 years ranging from of 24 to 38 years. No patient had history of pelvic endometriosis. A history of pelvic surgery involving the uterus was reported in all cases; caesarean section in twelve cases and three patients underwent myomectomy by laparotomy with intraoperative uterine cavity opening.

The main complaint was cyclic pelvic pain. It was reported in all cases and located at the level of abdominal scar. Two of them presented skin changes; in fact, they showed scar ecchymosis and hyperpigmentation in menstrual period. One patient had a history of skin bleeding throughout a scar fistula.

Clinical examination founds a palpable abdominal scar mass in all cases, measuring from 1,5 to 6 cm. In one case, a bleeding skin fistula was found.

14,8 months was the average time separating first symptom to surgery.

**Fig. 1:**
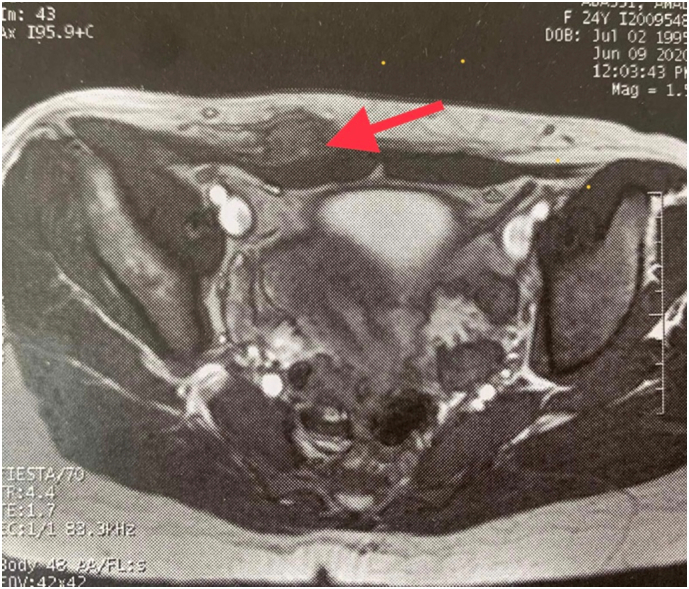
Abdominal wall endometriosis MRI signs: a hyperintense heterogeneous mass on both T1 and T2-weighted sequencies (red arrow). (For interpretation of the references to colour in this figure legend, the reader is referred to the web version of this article.)

Abdominal wall ultrasound and pelvic MRI were performed in all patients. Rectus abdominis muscle invasion was found in all cases (Fig. 1).

**Fig. 2:**
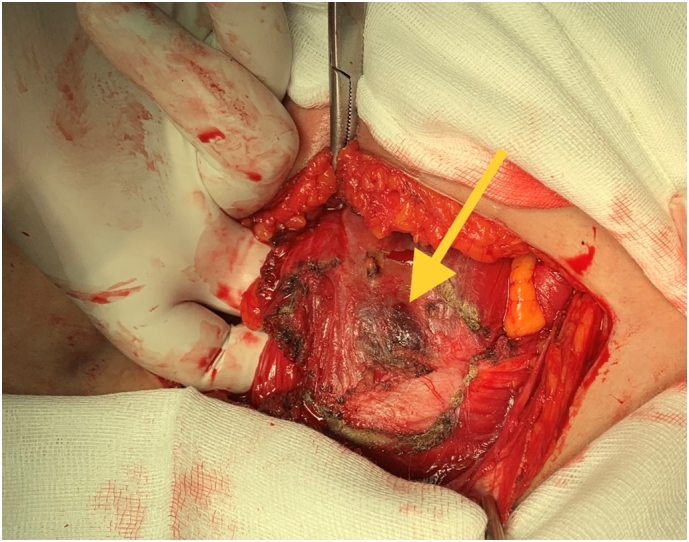
Rectus abdominis muscle endometriosis (yellow arrow). (For interpretation of the references to colour in this figure legend, the reader is referred to the web version of this article.)

**Fig. 3:**
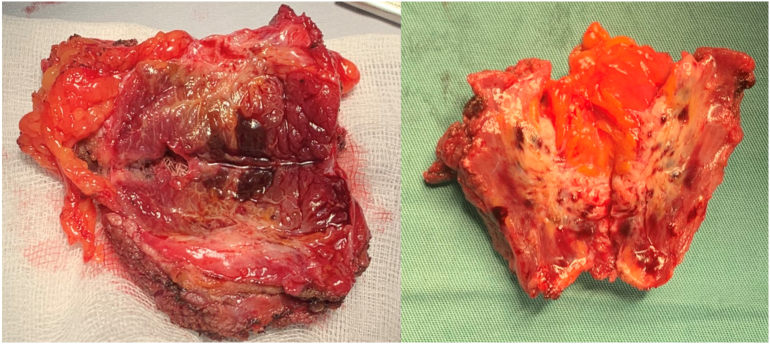
Surgical specimen of resected abdominal wall endometriosis, which shows a macroscopic specific endometriosis sign: the chocolate fluid.

Surgical excision of parietal endometriosis location was performed (Fig. 2, Fig. 3). Histologic examination confirmed abdominal wall endometriosis with clean surgical margins.

In one case, recurrence was reported 6 months after excision. The patient underwent a large excision with abdominoplasty to ensure abdominal wall closure and to ovoid abdominal hernia.

All procedures were uncomplicated. All patient's data are reported in Table 1.

**Table 1:** Clinical, imaging, surgical procedure and outcome features.

Case	Age (year)	Main symptom	Surgical history	Interval surgery symptoms (month)	Size (cm)	Imaging	Surgery	Follow-up
1	34	Cyclic pain	CS	17	2,0	US, MRI	SE	NR
2	29	Cyclic pain	CS	24	1,5	US, MRI	SE	NR
3	37	Cyclic pain	CS	13	3,0	US, MRI	SE	NR
4	38	Cyclic pain	2 CS	14	3,5	US, MRI	EMR	R
5	28	Cyclic pain	CS	9	2,0	US, MRI	SE	NR
6	24	Cyclic pain	Myomectomy	12	4,0	US, MRI	SE	NR
7	33	Cyclic pain	3 CS	7	2,5	US, MRI	SE	NR
8	35	Cyclic pain	CS	26	3,0	US, MRI	SE	NR
9	30	Cyclic pain	2 CS	18	7,0	US, MRI	EMR	NR
10	38	Cyclic pain	CS	12	3,0	US, MRI	SE	NR
11	29	Cyclic pain	Myomectomy	14	2,0	US, MRI	SE	NR
12	32	Cyclic pain	CS	13	3,5	US, MRI	SE	NR
13	31	Cyclic pain	CS	15	5,5	US, MRI	SE	NR
14	33	Cyclic pain	CS	17	4,5	US, MRI	SE	NR
15	29	Cyclic pain	Myomectomy	11	2,5	US, MRI	SE	NR

US = ultrasound, MRI = magnetic resonance imaging, SE = surgical excision, NR = no relapse, CS = caesarean section.

## 4 Discussion

Abdominal wall endometriosis is a rare clinical condition with unclear pathophysiology. The leading theory suggests that endometrial cells are implanted outside uterine cavity after gynaecologic manipulation as caesarean section or myomectomy. It occurs frequently after gynaecologic or obstetric surgery, reported incidence ranges from 0,03 to 3,5% [3]. In our study, two risk factors were incriminated: caesarean section and myomectomy. No patient had a history of pelvic endometriosis, it suggests that abdominal wall endometriosis and pelvic endometriosis haven't the same pathogenic mechanism and are different entities [4]. Abdominal wall endometriosis is caused mainly by iatrogenic spread of uterine cells in the abdominal wall layer [5]. Other generated hypothesis may explain parietal endometriosis location: lymphatic or hematogenous dissemination, metaplastic transformation and local immune cell changes [5], [6].

Esquivel and al described a clinical triad to suspect abdominal wall endometriosis [7]: history of open gynaecologic surgery, palpable abdominal mass and catamenial pain.

Ultrasonography, computed tomography and MRI are useful for diagnosis, specially to eliminate differential diagnosis: parietal abscess, lipoma, hematoma, hernia, granuloma and tumour [8].

Ultrasonography is the first step to evaluate painful abdominal wall mass [9]. Many informations can be provided: mass measurements, location, margins and structure.

In ultrasonography imaging, abdominal wall endometriosis appears as heterogeneous and hypoechoic mass with echogenic spots [10]. Margins may be blurred due to surrounding tumour inflammatory reaction.

MRI is the cornerstone technique to evaluate soft tissue tumours. In case of abdominal wall endometriosis, MRI is an interesting option to describe and evaluate mass size, extension, deep infiltration and involvement of surrounding structures [1], [11]. It is helpful to plan surgical management by predicting the parietal defect and evaluating the need of mesh parietal repair (abdominoplasty) [1].

In MRI, abdominal wall endometriosis appears as an hyperintense heterogeneous mass on both T1 and T2-weighted sequences.

The management of abdominal wall endometriosis is based on surgery with large excision. Margins must be clean at least 1 cm [12].

The surgery goals are to remove the mass and to confirm histologic diagnosis of parietal endometriosis.

For small nodule, surgery is easy to perform. With clean margins of 1 cm, recurrence rate is less than 5% [13]. Large tumour requires complex procedures including large resection and reconstructive parietal techniques such as muscle flaps and mesh parietal repair to ovoid recurrence and parietal defect. Positive margins, incomplete or inadequate resection are associated with high recurrence rate (9.1%) [14].

Surgery strategies should be planned after considering recurrence and parietal defect risks. For complex procedures, close coordination between gynecologic and visceral surgeons may guarantee better outcome.

Medical treatment based on oral contraceptives, analogues of gonadotropin-releasing hormone, aromatase inhibitors and dienogest may be useful if combined to surgical excision to avoid recurrence [15]. Non-steroidal anti-inflammatory agents are recommended to treat pain related to abdominal wall endometriosis location and to facilitate surgical excision by decreasing inflammation surrounding the lesions [16].

Abdominal wall endometriosis affects negatively social, sexual and professional activities, by generated pain, depression and absenteeism [14]. It can be considered as an iatrogenic and preventable damage.

Several methods in gynaecologic surgery are described to avoid abdominal wall endometriosis.

It is recommended to handle gently the uterine tissue, ensure meticulously the control of bleeding, wash of the intraabdominal cavity before closure and avoid subcutaneous dead spaces [17].

Randomised large studies are needed to define efficient methods to avoid abdominal wall endometriosis.

## 5 Conclusion

Parietal endometriosis is a rare clinical condition with unclear pathophysiology. Although rare, gynaecologists must be familiar with this pathology nowadays considering the high rate of caesarean section and gynaecological procedures. The diagnosis of abdominal wall endometriosis is based on clinical data, patient history, ultrasound and MRI. Surgical excision remains the cornerstone of treatment, it may require coordination with visceral surgeon in case of large and complex excision.

## Funding

No source of funding to declare.

## Ethical approval

Ethical approval of case series is not needed in our institution.

## Consent

Written informed consent was obtained form patients for publication of this case series and accompanying images. A copy of the written consent was available for review by the Editor-in-Chief of this journal on request.

## CRediT authorship contribution statement

Mohamed Aymen Ferjaoui and Ramzi Arfaoui: surgery and study design.

Slim Khedhri, Kais Abdessamia and Mohamed amine Hannechi: manuscript redaction.

Kmar Hajjami, Raja Azri and Manel Benghalia: data collection.

Monia Malek, Mohamed Radhouane Rachdi and Khaled Neji: Manuscript supervisors.

## Research registration

Not mandatory.

## Guarantors

Mohamed Aymen Ferjaoui and Ramzi Arfaoui.

## Provenance and peer review

Not commissioned, externally peer-reviewed.

## Declaration of competing interest

No conflicts of interest to report.
